# Coatomer complex I is required for the transport of SARS-CoV-2 progeny virions from the endoplasmic reticulum-Golgi intermediate compartment

**DOI:** 10.1128/mbio.03331-24

**Published:** 2024-11-29

**Authors:** Ai Hirabayashi, Yukiko Muramoto, Toru Takenaga, Yugo Tsunoda, Mayumi Wakazaki, Mayuko Sato, Yoko Fujita-Fujiharu, Norimichi Nomura, Koji Yamauchi, Chiho Onishi, Masahiro Nakano, Kiminori Toyooka, Takeshi Noda

**Affiliations:** 1 Laboratory of Ultrastructural Virology, Institute for Life and Medical Sciences, Kyoto University 12918 , Kyoto, Kyoto Prefecture, Japan; 2 CREST, Japan Science and Technology Agency 13501 , Kawaguchi, Saitama Prefecture, Japan; 3 Laboratory of Ultrastructural Virology, Graduate School of Biostudies, Kyoto University 12918 , Kyoto, Kyoto Prefecture, Japan; 4 RIKEN Center for Sustainable Resource Science 98319 , Yokohama, Kanagawa Prefecture, Japan; 5 Department of Cell Biology, Graduate School of Medicine, Kyoto University 12918 , , Kyoto, Kyoto Prefecture, Japan; 6 Institute for Integrated Cell-Material Sciences, Kyoto University 12918 , Kyoto, Kyoto Prefecture, Japan; Washington University in St. Louis School of Medicine, St. Louis, Missouri, USA; Johns Hopkins University, Baltimore, Maryland, USA

**Keywords:** SARS-CoV-2, COPI, vesicular transport

## Abstract

SARS-CoV-2 undergoes budding within the lumen of the endoplasmic reticulum-Golgi intermediate compartment (ERGIC), and the progeny virions are delivered to the cell surface via vesicular transport. However, the molecular mechanisms remain poorly understood. Using three-dimensional electron microscopic analysis, such as array tomography and electron tomography, we found that virion-transporting vesicles possessed protein coats on their membrane and demonstrated that the protein coat was coatomer complex I (COPI). During the later stages of SARS-CoV-2 infection, we observed a notable alteration in the distribution of COPI and ERGIC throughout the cytoplasm, suggesting their potential involvement in virus replication. Depletion of COPB2, a key component of COPI, led to the confinement of SARS-CoV-2 progeny virions within the ERGIC at the perinuclear region. While the expression levels of viral proteins within cells were comparable, this depletion significantly reduced the efficiency of virion release, leading to the significant reduction of viral replication. Hence, our findings suggest COPI as a critical player in facilitating the transport of SARS-CoV-2 progeny virions from the ERGIC. Thus, COPI could be a promising target for the development of antivirals against SARS-CoV-2.

**IMPORTANCE:**

SARS-CoV-2 virions are synthesized within the ERGIC and are transported to the cell surface via vesicular transport for release. However, the precise mechanisms remain unclear. Through various electron microscopic techniques, we identified the presence of COPI on virion-transporting vesicles. Alterations in the distribution of COPI and ERGIC in SARS-CoV-2 infected cells are evident, suggesting their involvement in virus replication. When COPB2, a component of COPI, is depleted, progeny virions become trapped within the ERGIC, leading to a reduction in the efficiency of virion release. These findings highlight COPI’s crucial role in mediating SARS-CoV-2 vesicular transport from the ERGIC and suggest it as a potential antiviral target.

## INTRODUCTION

Severe acute respiratory syndrome-coronavirus-2 (SARS-CoV-2), a member of the β-coronavirus family within *Coronaviridae*, is an enveloped RNA virus with a positive-stranded genome. It first emerged in late 2019 and has since spread rapidly worldwide, giving rise to various variants that adapt to human hosts and evade the immune system. Despite the development of effective vaccines that mitigate disease severity, coronavirus disease 2019 (COVID-19) continues to pose a significant threat to public health.

SARS-CoV-2 gains entry into host cells through the interaction between its spike protein (S) and the angiotensin-converting enzyme 2 (ACE2) receptor (1). Subsequent to the fusion of the viral envelope with either the plasma membrane or the endosomal membrane, following endocytosis (2), the positive-stranded RNA genome is released into the cytoplasm, initiating the synthesis of the RNA-dependent RNA complex (RdRp). RdRp transcribes negative-strand antigenomic RNAs, serving as templates for viral RNA genome replication and the production of sub-genomic mRNAs encoding viral proteins, such as S, membrane protein (M), envelope protein (E), and nucleocapsid protein (N), which collectively constitute the SARS-CoV-2 virus particles (3–5). Non-structural proteins 3, 4, and 6 (nsp3, nsp4, and nsp6, respectively) induce the formation of double-membrane vesicles (DMVs) (6–8), acting as sites for viral genome transcription and replication (9–11). Once all viral proteins are synthesized, viral assembly and the budding of progeny virions occur within the endoplasmic reticulum-Golgi intermediate compartment (ERGIC) (12–14), and these progeny virions are transported to the cell surface via vesicular transport and the biosynthetic secretory pathways (9, 15, 16). However, there is no consensus on the exit route or the origin of the transporting vesicles for progeny virions. It has been reported using mouse hepatitis virus that β-coronavirus is transported by large vesicles derived from an enlarged Golgi membrane, which is released due to the accumulation of progeny virions in the lumen (17). This suggests that SARS-CoV-2 virions assembled at the ERGIC enter the Golgi apparatus and are directed to the plasma membrane through the trans-Golgi network; however, this is questionable because the Golgi apparatus is extensively fragmented after β-coronavirus infection (12, 18, 19). A study using avian infectious bronchitis virus, a β-coronavirus, suggests that progeny virions are transported from the ERGIC to the plasma membrane via endocytic recycling endosomes (20), indicating that progeny virions bypass the fragmented Golgi apparatus. Ghosh et al. reported that β-coronaviruses, including SARS-CoV-2, employ lysosomes for virion transport instead of the biosynthetic secretory pathway (21). Mendonca et al. demonstrated that virions exit through tunnels connecting unidentified large virion-rich vesicles to the plasma membrane using cryogenic focused ion beam-scanning electron microscopy (cryo FIB-SEM) (19). Furthermore, Eymieux et al. employed serial-ultrathin section transmission electron microscopy (TEM) to show that virions are primarily transported and released through small secretory vesicles containing a single virion rather than large virion-rich vesicles (22). While these findings are not mutually exclusive due to the different experimental conditions such as variations in viruses, cell lines, and time points, the process of intracellular virion transport and the specific host factors involved in this process remain a subject of controversy and incomplete understanding (23).

To elucidate the intracellular transport mechanism of SARS-CoV-2 virions for their release, we initiated a comprehensive investigation of virus-infected cells. We initially performed a three-dimensional (3D) analysis of SARS-CoV-2-infected cells using scanning electron microscopy (SEM) and TEM. By employing immunostaining and gene knockdown techniques, we provide compelling evidence that coatomer complex I (COPI), which is responsible for retrograde vesicular transport from the Golgi apparatus to the endoplasmic reticulum (24–26) and is known to be involved in the transport of post-translationally modified S protein of β-coronavirus from the Golgi apparatus back to the ERGIC (27, 28), plays a crucial role in the transport of SARS-CoV-2 progeny virions from the ERGIC. Our findings provide new insights into a previously unrecognized function of COPI in SARS-CoV-2 replication.

## RESULTS

### Three-dimensional ultrastructural analysis of SARS-CoV-2-infected cells

**Fig 1:**
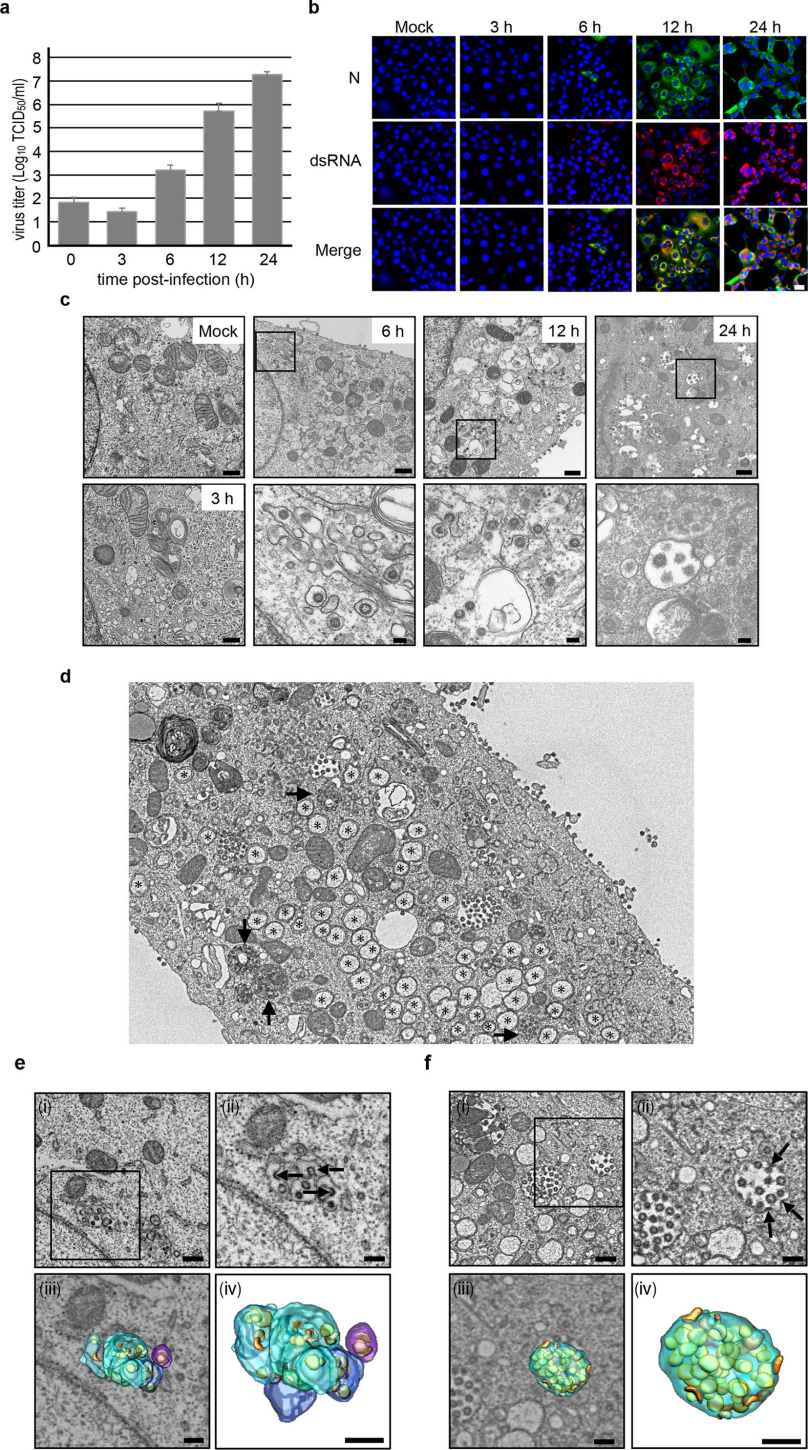
Replication kinetics of SARS-CoV-2 in VeroE6/TMPRSS2 cells. (a) Replication of SARS-CoV-2 in VeroE6/TMPRSS2 cells as determined by the 50% tissue culture infectious dose (TCID_50_) assay. The experiment was performed in biological triplicate. The error bars indicate the standard deviation (SD) of the three biological replicates. (b) Immunostaining images of mock- and SARS-CoV-2-infected VeroE6/TMPRSS2 cells using antibodies against the SARS-CoV-2 N protein (green) and double-stranded RNA (dsRNA) (red). Nuclei are stained with Hoechst 33342 (blue). The cells were infected with the virus at a multiplicity of infection (MOI) of 2 to collect the samples at 3 and 6 h post-infection (hpi) and at an MOI of 1 to collect samples at 12 and 24 hpi. Scale bar: 30 nm. (c) Ultrathin section images of SARS-CoV-2-infected VeroE6/TMPRSS2 cells at the indicated time points after infection. Box areas in images at 6, 12, and 24 hpi were magnified, respectively. Scale bars: 500 nm for low magnification images, and 100 nm for high magnification images. (**d–f**) Ultrastructure of endoplasmic reticulum-Golgi intermediate compartment (ERGIC) (e) and virion-containing vacuoles (f) in a virus-infected cell (d) imaged by scanning electron microscopy (SEM) array tomography. In (d), ∗ indicates double-membrane vesicles, and arrows indicate lysosomes. In (e) and (f), (i) Llow magnification SEM image and (ii) Hhigh magnification image of the box area in (i) are shown. Arrows in (e) and (f) indicate virus budding. (iii) 3D rendering of ERGIC vesicles/tubules (light blue, blue, and purple), virion-containing vacuoles (light green), budding virions (orange), and budded virions (yellow) wereas superimposed on the image shown in (ii). (iv) 3D rendering of ERGIC vesicles/tubules (light blue, blue, and purple), virion-containing vacuoles (light green), budding virions (orange), and budded virions (yellow) shown in (iii). Nuc,: nucleus. Scale bar: 400 nm (i), 200 nm (ii–iv).

**Fig 2:**
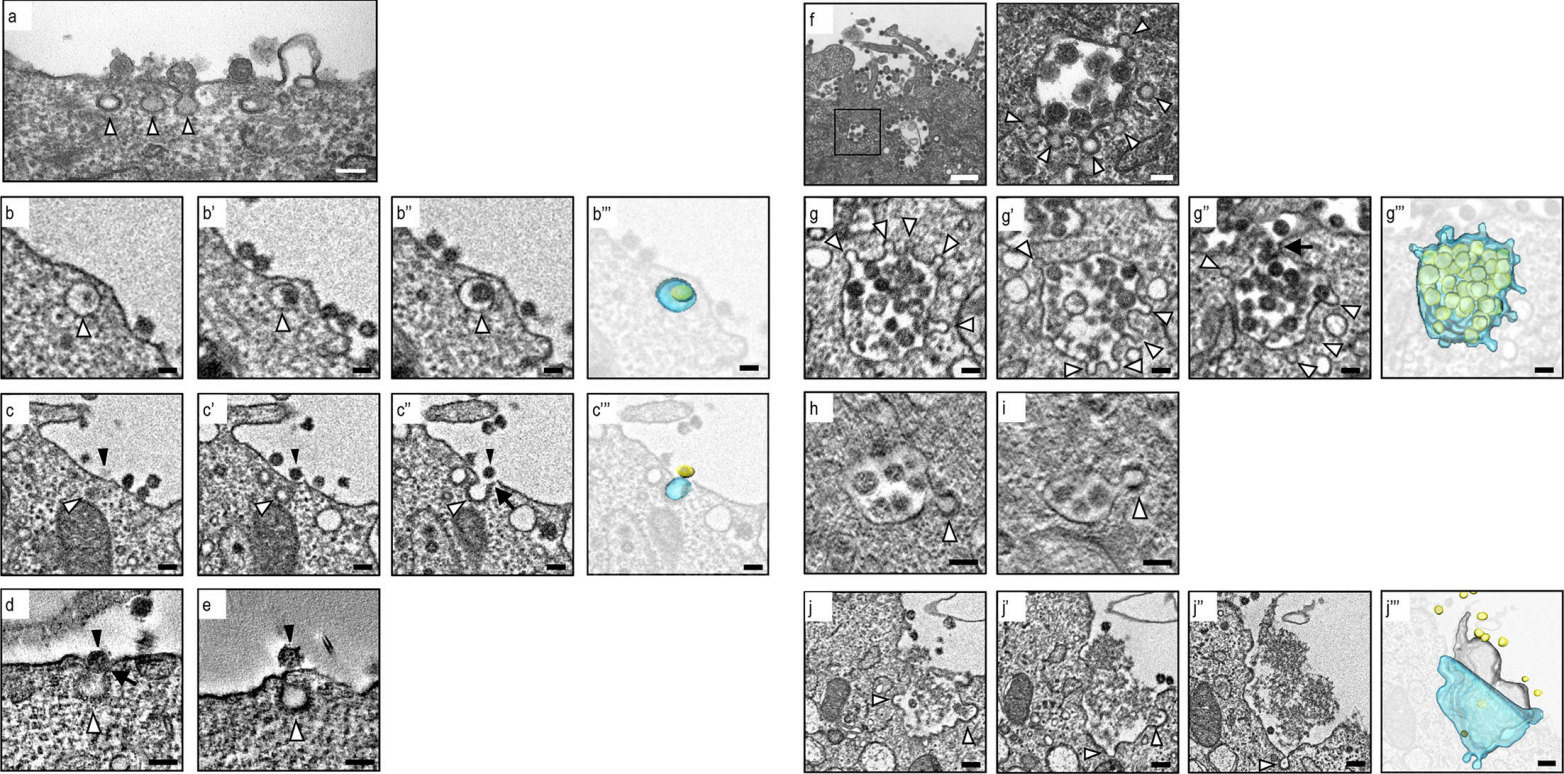
Ultrastructural analysis of SARS-CoV-2 virion transport using ultrathin-section TEM, SEM array tomography, and electron tomography. (a) An ultrathin-section image of the pits observed on the plasma membrane of a virus-infected cell. White arrowheads indicate pits close to SARS-CoV-2 virions. Scale bar: 100 nm. (**b and **c) Serial section images of the small vesicles containing single virions obtained using SEM array tomography. The b,b′, and b″ and c, c′, and c″ were taken from different sections of the same field, respectively. The b′″ and c′″ are superimposed images of b″ and c″ with 3D rendering of small vesicles (light blue) and virions (yellow), respectively. Black arrowheads indicate virions, and white arrowheads indicate small vesicle. The arrow indicates the membrane fusion between a vesicle and the plasma membrane. Scale bars: 100 nm. (**d and **e) Electron tomographic images of small vesicles transporting single virions. Black arrowheads indicate virions, and white arrowheads indicate coated proteins on the small vesicles. The arrow indicates the membrane fusion between a vesicle and the plasma membrane. Scale bars: 100 nm. (f) An ultrathin-section image of the multiple virions-containing large vacuoles near the plasma membrane. White arrowheads indicate membrane protrusion from the large vacuoles. Scale bars: 500 nm (left), 100 nm (right). (g) Serial section images of the large vacuole containing multiple virions obtained by SEM array tomography. The g, g′, and g″ were taken from different sections of The same field. The g′″ is a superimposed image of g″ with 3D rendering of a large vacuole (light blue) and virions (yellow). White arrowheads indicate membrane protrusions. Scale bars: 100 nm. (**h and i**) Electron tomographic images of large vacuoles transporting multiple virions. White arrowheads indicate coated proteins on the membrane protrusions. Scale bars: 100 nm. (j) Serial section images of the virion-containing lysosome obtained by SEM array tomography. The j, j′, and j″ were taken from different sections of the same field. The j′″ is a superimposed image of j″ with 3D rendering of the lysosome (light blue), virions (yellow), and degraded cellular components (gray). White arrowheads indicate membrane protrusions. Scale bars: 200 nm.

To investigate the growth kinetics of SARS-CoV-2, we infected VeroE6/TMPRSS2 cells with the virus at a multiplicity of infection (MOI) of 1 and monitored viral titers in the supernatants up to 24 h post-infection (hpi) using a 50% tissue culture infectious dose (TCID_50_) assay. Concurrently, we assessed the expression of the SARS-CoV-2 N protein and double-stranded RNA (dsRNA) as a marker for viral genome replication over time using immunofluorescence assays (IFA). Notably, viral replication became detectable at 6 hpi, with titers steadily increasing up to 24 hpi (Fig. 1a). In parallel, the expression of the N protein and dsRNA was observed in several cells at 6 hpi, with a subsequent increase in the number of virus-infected cells (Fig. 1b). Our examination using ultrathin-section TEM revealed the presence of single virions within vesicular and tubular structures, which morphologically corresponded to the endoplasmic reticulum-Golgi intermediate compartment (ERGIC) at 6 and 12 hpi, while multiple virion-containing vacuoles were conspicuous at 24 hpi (Fig. 1c). Therefore, for subsequent ultrastructural analysis, we employed samples collected at 24 hpi. We then embarked on a study of intracellular virion transport for release. Using SEM array tomography, we conducted a three-dimensional assessment of virion distribution within virus-infected cells (Video S1). This analysis unveiled various structures, including single virion-containing small vesicles, multiple virion-containing large vacuoles, and multiple virion-containing lysosomes dispersed within the cell, alongside numerous DMVs (Fig. 1d). Virions were also observed within the membranes of the ERGIC (Fig. 1e; Video S2) and large vacuoles during the budding process, which is consistent with prior reports (Fig. 1f; Video S3) (16). To investigate how the progeny virions are released into the extracellular space, we focused on the membranous structures underneath the plasma membrane. Ultrathin-section TEM revealed small pits in the plasma membrane, on which the virions were present (Fig. 2a). Given that the entry route of SARS-CoV-2 into cells, whether via the plasma membrane or endosomal pathway, is dependent on the cell type (1) and that the virus predominantly enters through the plasma membrane rather than via clathrin-mediated endocytosis in the presence of TMPRSS2 (29), it is considered that the virions observed near the coated pits are released virions. SEM array tomography affirmed that single virion-containing small vesicles were located beneath the plasma membrane (Fig. 2b) and connected to the plasma membrane (Fig. 2c) (Video S4). Notably, detailed ultrastructural analysis through TEM electron tomography revealed that the small vesicles/pits beneath the plasma membrane were coated with proteins (Fig. 2d and e; Video S5), suggesting virion release via exocytosis through these coated vesicles. Furthermore, ultrathin-section TEM exposed multiple virion-containing pleomorphic vacuoles near the plasma membrane, exhibiting several membrane protrusions forming a crown-like structure (Fig. 2f). SEM array tomography corroborated these findings, suggesting that pleomorphic vacuoles with membrane protrusions are connected to the plasma membrane (Fig. 2g; Video S6). TEM electron tomography revealed that the membrane protrusions on the pleomorphic vacuoles were also coated with proteins (Fig. 2h through j; Video S7), which is consistent with the observations in single virion-containing vesicles (Fig. 2d and e). These findings suggest that virions are released via exocytosis through large pleomorphic vacuoles with coated membrane protrusions. Coated membrane protrusions were also observed in virion-containing lysosomes fused to the plasma membrane (Fig. 2j; Video S8). In summary, our results indicate that while SARS-CoV-2 employs various vesicle types for virion transport, membrane-bound coat proteins likely play a crucial role in the vesicular transport of SARS-CoV-2 virions for their release.

### Involvement of COPI in the vesicular transport of SARS-CoV-2 virions

**Fig 3:**
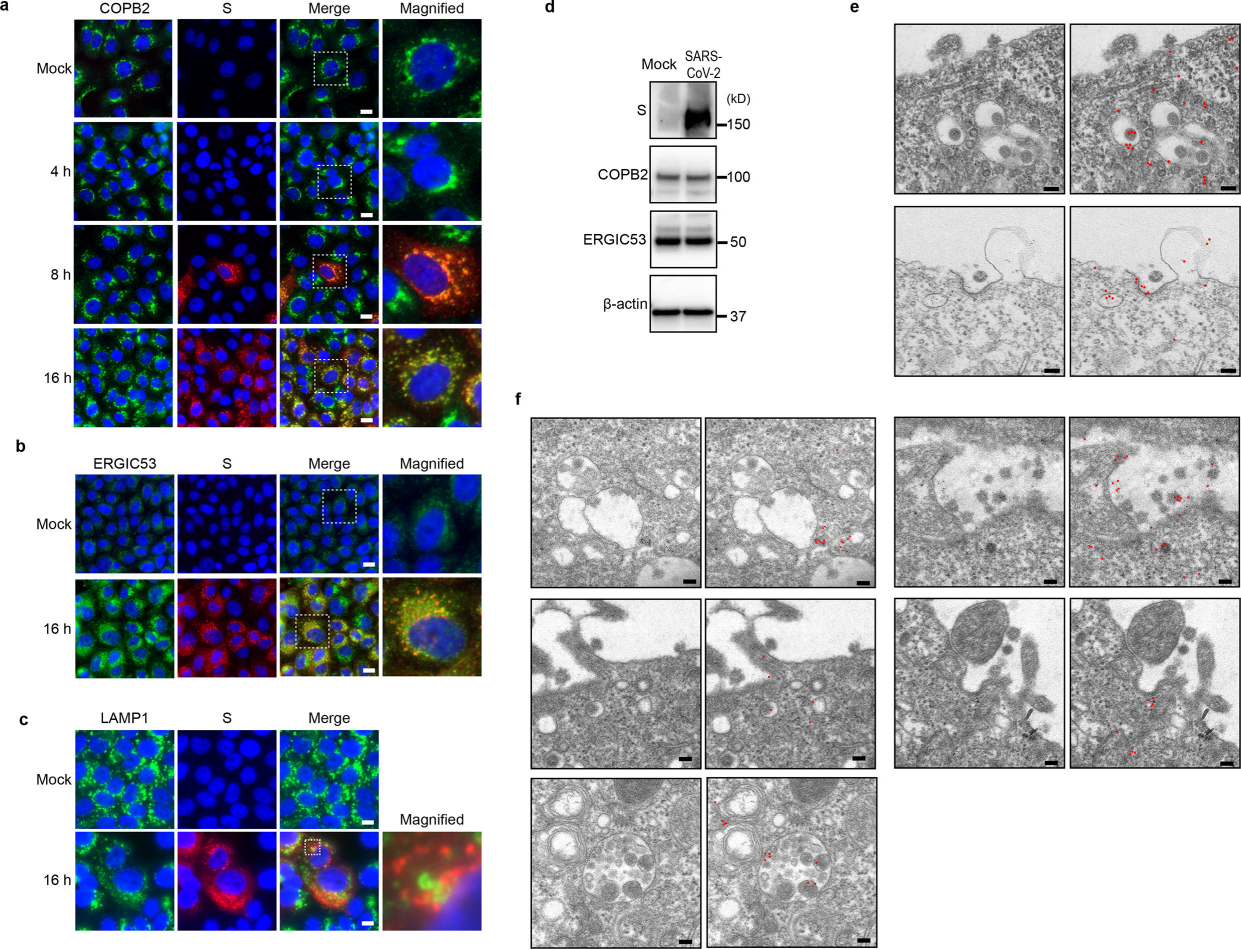
Intracellular localization of coatomer complex B2 (COPB2) and ERGIC53 in SARS-CoV-2-infected VeroE6/TMPRSS2 cells. (a–c) VeroE6/TMPRSS2 cells were infected with SARS-CoV-2 at an MOI of 1, fixed at the indicated time points, and subjected to an immunofluorescence assay using antibodies against COPB2 (green in a), ERGIC53 (green in b), LAMP1 (green in c), and SARS-CoV-2 S protein (red). Nuclei were stained with Hoechst 33342 (blue). Box areas shown in merged images are magnified and displayed in the right-hand column. Scale bars: 10 µm. (**D**) Western blot analysis showing the expression levels of SARS-CoV-2 S protein, COPB2, ERGIC53, and β-actin in mock-infected and SARS-CoV-2-infected cells at 16 hpi. (**E**) Immunoelectron microscopy of SARS-CoV-2-infected cells using antibodies against β-COP, followed by secondary antibody conjugated with 6 nm gold beads. (right images) For clarity, gold beads were labeled with red and are shown in the right column. Scale bars: 100 nm. (**F**) Immunoelectron microscopy of SARS-CoV-2-infected cells using antibodies against ERGIC53, followed by secondary antibody conjugated with 6 nm gold beads. For clarity, gold beads were labeled with red and are shown on the right side. Scale bars: 100 nm.

It is well known that clathrin, COPI, and COPII are major coatomers that associate with membranous vesicles, regulating vesicular transport (26, 30). Among these, COPI, which is composed of seven subunits, namely, α, β, β′, γ, δ, ε, and ζ, plays a critical role in protein-transporting vesicles responsible for retrograde transport from the ERGIC (24–26, 31), the primary sites for SARS-CoV-2 assembly and budding (12–14, 32). Hence, we postulated that the membrane-bound coat proteins found on vesicles and vacuoles containing virions are COPI, and COPI is involved in the transport of progeny virions from the ERGIC. To test this hypothesis, we examined the localization of β-COP or COPB2, subunits of the COPI complex, and ERGIC53, a specific ERGIC marker, in SARS-CoV-2 infected cells using IFA and immunoelectron microscopy (IEM). In mock-infected cells, COPB2 was primarily localized in the perinuclear region. However, during the later stages of SARS-CoV-2 infection, its distribution changed (Fig. 3a). At 8 hpi, COPB2 colocalized with the SARS-CoV-2 S protein in the perinuclear region. By 16 hpi, both COPB2 and the SARS-CoV-2 S protein exhibited a punctate pattern throughout the cytoplasm (Fig. 3a). In contrast to mock-infected cells, ERGIC53 exhibited a punctate distribution in the cytoplasm at 16 hpi and colocalized with the SARS-CoV-2 S protein (Fig. 3b). Western blot analysis indicated that ERGIC53 expression in virus-infected cells was similar to that in mock-infected cells (Fig. 3d), suggesting that SARS-CoV-2 replication may cause membrane alteration or fragmentation of the ERGIC. Unlike a previous report, LAMP1, a lysosome marker, exhibited only slight colocalization with the S protein (Fig. 3c) (11). IEM revealed that both β-COP and ERGIC53 were detected adjacent to virion-containing vesicles in the cytoplasm, virion-containing vesicles near the plasma membrane, and coated pits at the plasma membrane (Fig. 3e and f). Statistical analysis of the β-COP and ERGIC-53 signals adjacent to virion-containing vesicles/coated pits near the plasma membrane of infected cells and those located near the plasma membrane of uninfected cells showed that the signals for both β-COP and ERGIC-53 adjacent to virion-containing vesicles/coated pits near the plasma membrane were detected at significantly higher frequencies than vesicles/coated pits near the plasma membrane in uninfected cells (Table 1). It suggests that β-COP and ERGIC53 are translocated to the plasma membrane along with virion-containing vesicles. Collectively, these findings support the notion that COPI plays a role in intracellular virion transport through ERGIC-derived vesicles.

**TABLE 1:** Immuno-EM signals adjacent to vesicles and coated pits near the plasma membrane

	Category	Gold signal					*χ* ^2^	*P* value
		Positive		Negative		Total count		
		Count	%	Count	%			
β-COP	Vesicles and coated pits in uninfected cells	5	10	45	90	50	44.43	<0.00001
	Virion-containing vesicles and coated pits in infected cells	38	76	12	24	50		
ERGIC-53	Vesicles and coated pits in uninfected cells	5	10	45	90	50	41.50	<0.00001
	Virion-containing vesicles and coated pits in infected cells	37	74	13	26	50		

### COPI facilitates the release of virion-containing vesicles from the ERGIC

**Fig 4:**
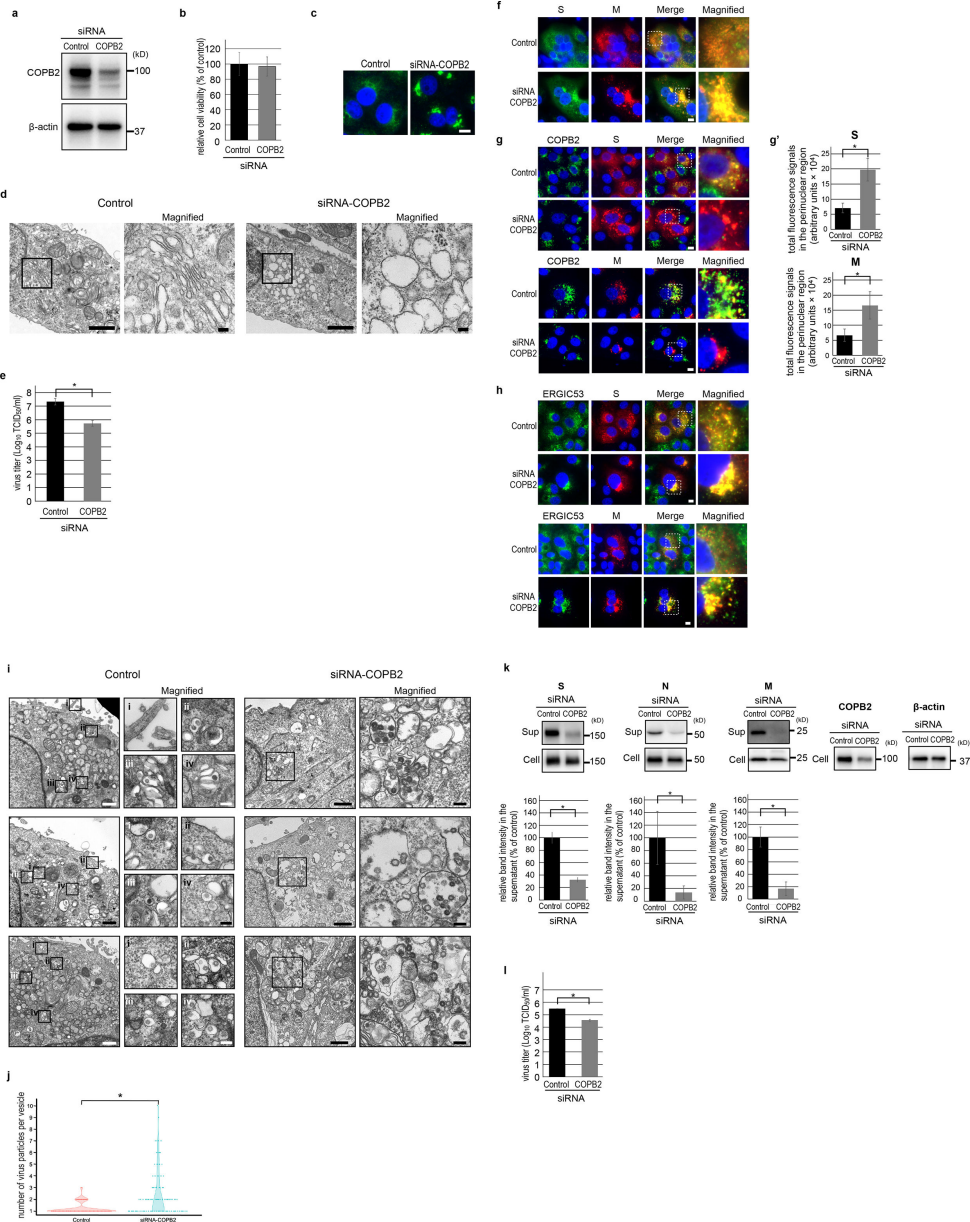
Impact of COPB2 depletion on SARS-CoV-2 replication. (a) The expression level of COPB2 in siRNA-treated Vero E6 cells was examined at 24 h post-transfection by western blotting. (b) Cell viability was evaluated using a CellTiter-Glo assay at 24 h post-transfection. Error bars indicate the standard deviation (SD) of three biological replicates. (c) Vero E6 cells were transfected with a control siRNA or siRNA against COPB2 and fixed at 24 h post-transfection. COPB2 and ERGIC53 were immunolabeled, respectively. Nuclei were stained with Hoechst 33342 (blue). Bar: 10 µm. (d) Ultrathin-section TEM images of control and COPB2-KD cells at 24 h post-transfection. Scale bars: 1 µm (low magnification images) and 100 nm (high magnification images). (**E**) Virus titers in the supernatants of COPB2-KD and control cells at 24 hpi. Error bars indicate the SD of three biological replicates. A two-tailed Student’s *t*-test was used to compare the two groups. *P < 0.05. (f–h) Vero E6 cells were transfected with a control siRNA or siRNA against COPB2, infected by SARS-CoV-2, and fixed at 16 hpi. COPB2 (green in g), ERGIC53 (green in h), SARS-COV-2 S protein (green in f, red in g and h), and SARS-CoV-2 M protein (red in f–h) were immunolabeled. Nuclei were stained with Hoechst 33342 (blue). Bars:10 µm. (g′) The graphs display total intensities of S and M proteins around the nuclei of virus-infected control and COPB2-KD cells. Twenty virus-infected cells were randomly chosen, and the arbitrary units were calculated by the sum of the multiplication of each intensity by the areas. Error bars indicate the SD of three biological replicates. A two-tailed student’s *t*-test was used to compare the two groups. *P < 0.05. (i) Ultrathin-section TEM images of control and COPB2-KD cells infected with SARS-CoV-2 at 16 hpi. Control: Respective box regions (i**–iv**) are magnified and shown on the right. Scale bars: 1 µm (low magnification images) and 100 nm (high magnification images). siRNA-COPB2: Box regions are magnified and shown on the right. Scale bars: 1 µm (low magnification images) and 100 nm (high magnification images). (j) Quantification of the number of virions within the vesicles. A hundred virion-containing vesicles in both control and COPB2-KD infected cells were randomly selected, and the number of virions present within each vesicle was counted. A two-tailed Student’s *t*-test was used to compare the two groups. *P < 0.0001. (k) Detection of viral structural proteins in virus-infected control and COPB2-KD cells by western blot analysis. The experiment was performed in triplicate, and the band intensities of respective viral proteins were quantified and are shown as respective graphs. Error bars indicate the SD of three biological replicates. A two-tailed student’s *t*-test was used to compare the two groups. *P < 0.05. (l) Virus titers in the supernatants of COPB2-KD and control cells at 8 hpi. Error bars indicate the SD of three biological replicates. A two-tailed student’s *t*-test was used to compare the two groups. *P < 0.05.

To evaluate the significance of COPI in SARS-CoV-2 virion transport, we downregulated COPB2 expression using COPB2-targeting siRNAs. We confirmed a 50% reduction in COPB2 expression in COPB2-knockdown (COPB2-KD) cells compared to control cells without affecting cell viability (Fig. 4a and b). Immunostaining of uninfected COPB2-KD cells showed that ERGIC53 accumulated at the perinuclear region (Fig. 4c), where many ERGIC-like vacuoles were observed using ultrathin section TEM (Fig. 4d). COPB2 depletion significantly reduced the titer of infectious virions released into the extracellular medium to 2.5% at 24 hpi (Fig. 4e), underscoring the essential role of COPI in efficient SARS-CoV-2 replication. To assess the impact of COPI on the vesicular transport of SARS-CoV-2 virions, we examined the intracellular localization of viral proteins in COPB2-KD cells using IFA. In control infected cells at 16 hpi, the SARS-CoV-2 S and M proteins, both of which are structural proteins of the virion, exhibited colocalization (Fig. 4F) and were broadly distributed throughout the cytoplasm. In contrast, COPB2-KD infected cells exhibited altered distribution patterns for these viral proteins, with significantly increased signals in the perinuclear region (Fig. 4f, g and g’). Additionally, the localization of ERGIC53 in COPB2-KD cells was constrained to the perinuclear region, where the SARS-CoV-2 S and M proteins colocalized, indicating the accumulation of virions in this region (Fig. 4h). Ultrathin-section TEM revealed that ERGIC-like vacuoles containing virions were mainly limited to the perinuclear region in COPB2-KD infected cells, whereas virion-containing vesicles were dispersed throughout the cytoplasm in control infected cells at the same time point (Fig. 4i). Importantly, the number of virions within the vacuoles in COPB2-KD infected cells was significantly higher than that in control cells (Fig. 4j), suggesting that the depletion of COPB2 impairs the transport of virion-containing vesicles from the ERGIC. Lastly, to gauge the impact of suppressing COPI vesicular transport on extracellular virion release, we examined the amounts of viral S, N, and M proteins in the cells and released into the supernatant at 8 hpi, which corresponds to a single replication cycle of the virus (Fig. 4k). Additionally, we examined virus titers at the same time point (Fig. 4l). Although COPB2-KD infected cells exhibited comparable viral protein levels to control infected cells, the relative amounts of viral protein in the supernatants of COPB2-KD infected cells were significantly reduced to 15%–25% compared to control infected cells (Fig. 4k). Consistently, the virus titer detected in the supernatant of COPB2-KD infected cells was reduced to approximately 11% compared to that in the supernatant of control infected cells (Fig. 4l). These results confirm the necessity of COPI vesicular transport for virion release from infected cells. In summary, these findings underscore the pivotal role of COPI in facilitating the transport of virions from the ERGIC, thereby enabling their release from infected cells.

## DISCUSSION

Progeny virions of SARS-CoV-2, which bud into the lumen of the ERGIC, necessitate transport through membranous structures to exit the cell. Yet, the molecular mechanisms governing this process have remained elusive. In this study, we conducted a three-dimensional ultrastructural analysis of virus-infected cells using SEM array tomography and electron tomography and discovered that the vesicles containing virions were outfitted with membrane coat proteins. Immunostaining revealed that the coat protein in question was COPI, and these virion-containing vesicles originated from the ERGIC. Crucially, COPI depletion hindered the transport of virions from the ERGIC, resulting in an accumulation of the progeny virions within the ERGIC, a notable reduction in virion release, and a decrease in viral replication. Collectively, our findings revealed the pivotal role of COPI during the SARS-CoV-2 life cycle.

During retrograde transport from the Golgi apparatus and ERGIC to the ER, the COPI complex plays a central role in cargo sorting, cargo uptake, vesicle formation, and vesicular release from membranes, giving rise to COPI-coated cargo-transporting vesicles (33). COPI interacts with a cytosolic dilysine motif, K-X-K-X-X and K-K-X-X (where X is any amino acid) in cargo proteins for their transport through the WD40 domains of α and β‘ COPI subunits (26, 34). In a previous kinome-wide siRNA screening for SARS-CoV, COPI was identified as a proviral host factor although its precise role remains unknown (35). Several studies have also reported the utilization of the COPI complex for transporting post-translationally modified S proteins from the Golgi apparatus back to the ERGIC for virion assembly during SARS-CoV and SARS-CoV-2 replication. This transport is mediated through a dibasic motif in the cytoplasmic tail of the S protein, K-X-H-X-X (where X can be arbitrary amino acid) (27, 28, 36, 37), although it is sub-optimal due to the substitution with His in the canonical motif. In our investigation, we unearthed an additional role of COPI, one that involves the intracellular transport of progeny virions. It appears that COPI is indispensable for the formation of vesicles that contain virions on the ERGIC membrane, thereby facilitating the transport of progeny virions from the ERGIC. Because the progeny virions within the ERGIC cannot interact with the COPI component due to their opposite topology, we suppose that SARS-CoV-2 merely takes advantage of this vesicular transport system regulated by the COPI complex to transport progeny virions from the ERGIC, which differs from the retrograde transport mechanism of the S protein specifically regulated by the COPI complex. Intriguingly, although COPI coats typically dissociate from vesicles shortly after their release during retrograde transport, they continued to be observed on many vesicles containing virions, even after transport to the cell surface. Future studies should explore whether COPI associated with virion-containing vesicles plays additional roles in intracellular transport and/or the fusion process at the plasma membrane for virion release.

**Fig 5:**
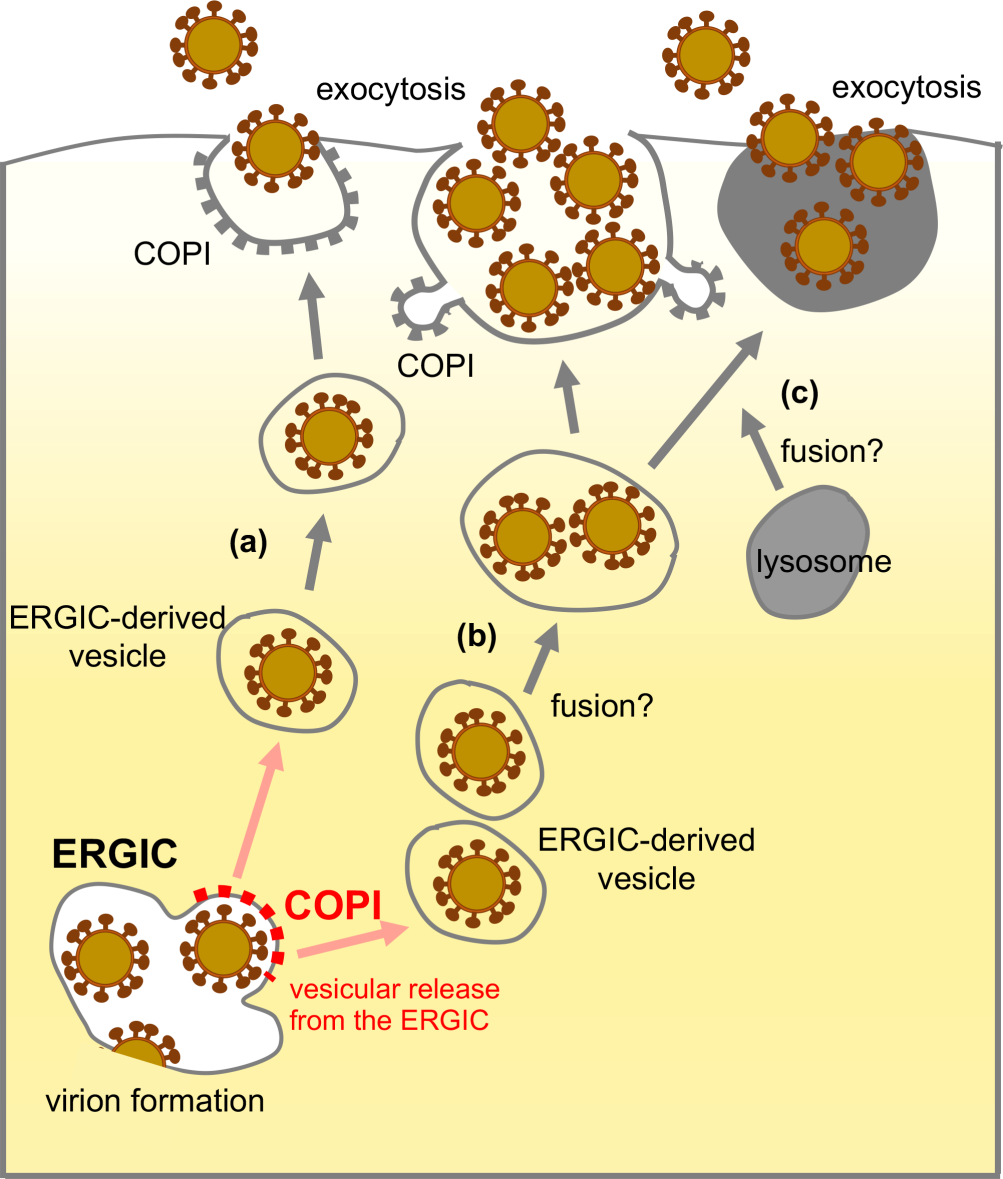
A proposed model of the vesicular transport of SARS-CoV-2. Virions bud into the lumen of the ERGIC. (**A**) Thereafter, single virion-containing vesicles are released from the ERGIC, which is mediated by the COPI complex. (**B**) Many single virion-containing vesicles are released from the ERGIC as the budding efficiency of virions increases during the later stages of infection. During their transport to the cell surface, multiple virion-containing vesicles may be formed by the repetitive fusion of virion-containing vesicles. (**C**) Virion-containing vesicles may be fused with lysosomes during their transport to the cell surface. Red dots on the ERGIC represent COPI.

A recent study identified lysosomes as vesicular transporters of progeny virions (21). Additionally, it has been suggested that progeny virions are transported through single- (22) and multiple-virion-containing vesicles (19) although the origins of these vesicles remain unidentified (23). In our ultrastructural analysis, single-virion-containing vesicles were predominantly observed at 6 and 12 hpi, whereas multiple-virion-containing vesicles and virion-containing lysosomes were more frequent at 24 hpi. This suggests that these vesicular transport systems are not mutually exclusive and that the vesicles observed in infected cells depend on the timing of viral infection and replication. Thus, based on our findings and in conjunction with previous reports, we propose a hypothetical model where the vesicular transport of virions from the ERGIC is mediated by COPI (Fig. 5). During the early stages of viral infection, the efficiency of progeny virion formation is relatively low due to the low expression of viral proteins and the viral genome. Consequently, a single virion buds into the lumen of the ERGIC, and subsequently, single virion-containing vesicles are released from the ERGIC, which is mediated by the COPI complex (Fig. 5A). These single virion-containing vesicles are then individually transported to the cell surface, probably bypassing the Golgi apparatus in most cases, because only a small number of single virions are found within the Golgi apparatus rims and most virions are observed in small cytoplasmic vesicles. As viral expression levels increase during the later stages of infection, the efficiency of progeny virion formation also increases, resulting in the budding of multiple virions into the ERGIC lumen. Consequently, many single-virion-containing vesicles are efficiently and simultaneously released from the ERGIC by the COPI complex, which may lead to the fusion of these vesicles to form larger vesicles during the transport of virions to the cell surface (Fig. 5B). Alternatively, multiple virion-containing vesicles may be released from the ERGIC although it is unclear whether the COPI complex has the ability to form large vesicles containing multiple virions. Because virion-rich Golgi apparatuses were never observed in our study and intracellular membranous structures including the Golgi apparatus are known to be fragmented and reorganized after SARS-CoV-2 infection (12, 19, 38), it is likely that multiple virion-containing vesicles bypass the Golgi apparatus and are directed directly to the plasma membrane. Our analysis did not reveal budding virions at the lysosomal membrane; thus, it is presumed that lysosomes may fuse with virion-containing ERGIC-derived vesicles during their transport to the cell surface (Fig. 5C). Therefore, we propose that regardless of their size, virion-containing vesicles primarily originate from the ERGIC, and their transport from the ERGIC is regulated by the COPI complex. Considering the fragmentation of the Golgi apparatus during SARS-CoV-2 replication, virion-containing vesicles are likely transported independently of the Golgi apparatus although some virions can be transported through the Golgi apparatus before the Golgi apparatus is fragmented (32, 39).

In summary, our study has demonstrated that COPI plays a critical role in the vesicular transport of SARS-CoV-2 virions from the ERGIC. This work unveils a novel role of COPI in the SARS-CoV-2 life cycle and underscores its significance in replication. Given its involvement in multiple steps, including virion transport from the ERGIC and retrograde transport of the S protein during viral replication, COPI represents a potential target for novel antiviral development.

## MATERIALS AND METHODS

### Cells and virus

Vero E6 and VeroE6/TMPRSS2 cells (40) were maintained in Dulbecco’s modified Eagle’s medium (DMEM) containing 10% fetal calf serum at 37°C under 5% CO_2_. The SARS-CoV-2 isolate (SARS-CoV-2/Hu/DP/Kng/19-027), provided by the Kanagawa Prefectural Institute of Public Health, was propagated in VeroE6/TMPRSS2 cells, and titers were determined by TCID_50_. All SARS-CoV-2 experiments were performed in a biosafety Level 3 containment laboratory at the Institute for Life and Medical Sciences, Kyoto University.

### Antibodies

The commercially available primary antibodies used for immunofluorescence, western blotting, and immuno-electron microscopy were as follows: anti-dsRNA mouse monoclonal antibody J2 (10010200; Scicons; Nordic MUbio, Susteren, Netherlands), anti ERGIC-53 Polyclonal antibody (13364-1-AP, proteintech, IL, USA), anti-β-COP rabbit polyclonal antibody (ab2899; abcam, UK), anti-COPB2 rabbit polyclonal antibody (A304-523A; Bethyl, Montogomery, TX, USA), (ab192924; abcam, UK), anti-SARS-CoV-2-S rabbit polyclonal antibody (NB100-56578; Novus Bililigicals, Centennial, CO, USA), anti-SARS-CoV-2-N rabbit polyclonal antibody (GTX135357; GeneTex, Irvine, CA, USA), anti-SARS-CoV-2-N mouse monoclonal antibody (ZMS1075; Merck, Darmstadt, Germany), anti-LAMP1 rabbit polyclonal antibody (9091T; Cell Signaling Technology, Danvers, MA, USA), anti-Clathrin hevy chain antibody (ab21679, abcam, UK), and anti-β-actin mouse monoclonal antibody (ab8226; abcam, UK). In-house anti-SARS-CoV-2-S mouse monoclonal antibody (#7), anti-SARS-CoV-2-M mouse monoclonal antibodies (YN7730-01), and mouse antiserum against SARS-CoV-2 M (#27-1-3) were generated by immunizing mice with the purified receptor-binding domains of the SARS-CoV-2 S and SARS-CoV-2-M proteins, respectively. The secondary antibodies used were Alexa Fluor 488-conjugated anti-rabbit (A11008; Thermo Fisher Scientific, Waltham, Ma, USA), Alexa Fluor 555-conjugated anti-mouse (A21422; Thermo Fisher Scientific), horseradish peroxidase-conjugated anti-mouse (NA931; GE Healthcare, Chicago, IL), anti-rabbit (NA934; GE Healthcare), and 6 nm gold-conjugated anti-rabbitantibody (711-195-152; Jackson ImmunoResearch, West Grove, PA, USA).

### Virus titration

The viruses were titrated using the TCID_50_ assay, as described previously (41). Briefly, VeroE6/TMPRSS2 cells were seeded in a 96-well plate 1 day prior to the assay. Serially diluted virus samples were inoculated into the cells and incubated at 37°C for 4 days. Thereafter, the cells were observed under a microscope to assess cytopathic effects. The viral titer (TCID_50_/mL) was calculated using the Reed–Muench method (42).

### Immunofluorescence assay

VeroE6 or VeroE6/TMPRSS2 cells infected with SARS-CoV-2 were fixed with 4% paraformaldehyde at 4°C for 1 h and permeabilized with 0.1% Triton X-100 for 10 min. After blocking with Blocking One solution (Nacalai Tesque) for 30 min, the cells were treated with antibodies against the SARS-CoV-2 N protein (1:1,000 dilution, GTX135357, ZMS1075), dsRNA (1:500 dilution, 10010200), COPB2 (1:1,000 dilution, ab192924), SARS-CoV-2-S (1:200 dilution, #7), (1:100 dilution, NB100-56578), or SARS-CoV-2-M (1:100 dilution, YN7730-01), followed by secondary antibodies against rabbit and mouse IgG. Nuclei were stained with Hoechst 33342 (1:1,000 dilution, H3570, Thermo Fisher Scientific). Fluorescent images were obtained using a BZ-X800 fluorescence microscope (Keyence, Japan). For quantification of the total fluorescence signals, the signals corresponding to S and M proteins around the periphery of the nuclei were automatically examined. Initially, a mask was created by extending about 4 µm from the nuclear boundary of each cell, followed by a smoothing process applied to the mask. Then, the nuclear area was subtracted from the masked area to quantify the signals only around the nuclear periphery. The total signal within the mask was calculated by multiplying the signal area of viral proteins by the intensities within the masked area. All data were analyzed using BZ-X800 Analyzer (Keyence, Japan).

### Sample preparation for electron microscopy

Vero E6 or VeroE6/TMPRSS2 cells infected with SARS-CoV-2 were fixed with 2.5% glutaraldehyde in 0.1 M cacodylate buffer on ice for 1 h and postfixed with 1% osmium tetroxide on ice for 1 h. The fixed samples were dehydrated using a series of ethanol gradients, substituted with propylene oxide, and embedded in epoxy resin.

### Ultrathin-section TEM, electron tomography, and immunoelectron microscopy

The epoxy blocks were trimmed, and ultrathin sections (~50 nm) were cut using an ultramicrotome (EM UC7; Leica, Wetzlar, Germany) equipped with an ultra-diamond knife (Diatome, Biel, Switzerland). For electron tomography, ultrathin sections (~200 nm) were cut, stained with 2% uranyl acetate and Reynold’s lead, and coated with carbon on both sides. The tilt series were recorded with a 200-kV TEM (TalosF200C, FEI, Thermo Fisher Scientific), and the tomograms were reconstructed using the simultaneous iterative reconstruction technique using IMOD (43). For immunoelectron microscopy, ultrathin sections were prepared on nickel grids, as described above, and incubated with saturated sodium periodate solution, followed by 0.2 M glycine in phosphate-buffered saline (PBS) buffer (44). After washing with PBS, sections were incubated with blocking buffer and either anti-β-COP antibodies (1:100 dilution, ab2899) or anti-ERGIC-53 antibody (1:40 dilution, 13364-1-AP). Thereafter, sections were washed with PBS and incubated with anti-rabbit immunoglobulin conjugated to 6 nm gold particles (1:30 dilution, 711-195-152). Ultrathin sections were stained with uranyl acetate and lead citrate and observed in an HT-7700 (Hitachi High-Tech Corporation, Japan) at 80 kV. For the statistical analysis of β-COP and ERGIC-53 signals adjacent to vesicles and coated pits, we counted the number of signal-positive and negative virion-containing vesicles/coated pits near the plasma membrane in more than 35 infected cells (within approximately 0.5 µm of the plasma membrane) and vesicles/coated pits near the plasma membrane in more than uninfected cells (within approximately 0.5 µm of the plasma membrane). Then, the data were subjected to statistical analysis using the chi-squared test.

### SEM array tomography

The epoxy blocks were trimmed, and serial sections (30 nm thick) were cut with an ultramicrotome (EM UC7) using an ultra-diamond knife (Diatome). Sections were mounted on silicon wafers. Serial sections were stained with uranyl acetate and lead citrate and coated with osmium coater (HPC-SW; Vacuum Device Co.). Images were obtained using field-emission SEM equipped with an auto capture for array tomography system (Regulus8220 ACAT; Hitachi High-Tech Corporation, Tokyo, Japan) with a backscattered electron detector at 2 kV. Three-dimensional image reconstruction was performed using Image-Pro software (Hakuto, Tokyo, Japan).

### siRNA treatment

Three siRNAs targeting COPB2 (# 9276-1, # 9276-2, # 9276-3) were purchased from Bioneer Corporation (USA). AllStars Negative Control siRNA (# 1027280; GIAGEN, Hilden, Germany) was used as a negative control. Vero E6 cells cultured in 24-well plates were transfected with a mixture of 25 nM siRNAs using the IT-X2 reagent (Mirus Bio LLC, USA). A second transfection was performed 24 h after the first transfection, which was subjected to viral infection 1 day after the second transfection. The downregulation of COPB2 was evaluated by western blotting.

### Cell viability assay

To evaluate cell viability, cells were collected 24 h after siRNA treatment, and the amount of intracellular ATP was measured using the CellTiter-Glo assay according to the manufacturer’s protocol (Promega, Madison, WI, USA).

### Western blotting

Virus-infected cells and the supernatants were dissolved with Tris-glycine SDS sample buffer (Thermo Fisher Scientific, Waltham, MA, USA), boiled for 5 min in the absence of reducing agent, and subjected to sodium dodecyl sulfate-polyacrylamide gel electrophoresis (SDS-PAGE). The proteins were electroblotted onto Immobilon-P transfer membranes (Merck). Thereafter, membranes were blocked with Blocking One for 30 min at room temperature and then incubated with primary antibodies, anti-COPB2 antibody (1:5,000 dilution), anti-SARS-CoV-2 S protein (1:1,000 dilution, NB100-56578), anti-SARS-CoV-2 N protein (1:10,000, GTX135357), a mouse antiserum against SARS-CoV-2 M protein (1:1 dilution), or anti-β-actin antibody (1:10,000 dilution) overnight at 4°C. After incubation with horseradish peroxidase-conjugated secondary antibodies (1:10,000 dilution) for 1 h at room temperature, the blots were developed using Chemi-Lumi One Super (Nacalai Tesque).

### Statistical analysis

Statistically significant differences in cell viability, virus titers, and the relative amounts of viral proteins were determined using a two-tailed student’s *t*-test. For viral titers, the statistical tests were performed on the log_10_ transformed data. In the figures, asterisks denote statistical significance as calculated by the Student’s *t*-test (*P < 0.05). The error bars indicate the standard deviation (SD).

## SUPPLEMENTAL MATERIAL

Supplemental Legends mbio.03331-24-s0001.docx Legends for Movies S1 to S8.

Movie S1 mbio.03331-24-s0002.mp4 3D image of a SARS-CoV-2-infected VeroE6/TMPRSS2 cell reconstructed using SEM array tomography.

Movie S2 mbio.03331-24-s0003.mp4 3D image of ERGICs containing progeny virions reconstructed using SEM array tomography.

Movie S3 mbio.03331-24-s0004.mp4 3D image of a multiple-virion-containing large vacuole reconstructed using SEM array tomography.

Movie S4 mbio.03331-24-s0005.mp4 3D image of single virion-containing small vesicles reconstructed using SEM array tomography.

Movie S5 mbio.03331-24-s0006.mov Electron tomographic image of small vesicles/pits coated with proteins that are located beneath the plasma membrane of virus-infected cell.

Movie S6 mbio.03331-24-s0007.mp4 3D image of a multiple-virion-containing large vacuole located beneath the plasma membrane of the virus-infected cell, reconstructed using SEM array tomography.

Movie S7 mbio.03331-24-s0008.mov Electron tomographic image of multiple-virion-containing vesicles located beneath the plasma membrane of virus-infected cell.

Movie S8 mbio.03331-24-s0009.mp4 3D image of a multiple-virion-containing lysosome fused with the plasma membrane of virus-infected cell, reconstructed using SEM array tomography.

ASM does not own the copyrights to Supplemental Material that may be linked to, or accessed through, an article. The authors have granted ASM a non-exclusive, world-wide license to publish the Supplemental Material files. Please contact the corresponding author directly for reuse.

## References

[1] Hoffmann M, Kleine-Weber H, Schroeder S, Krüger N, Herrler T, Erichsen S, Schiergens TS, Herrler G, Wu NH, Nitsche A, Müller MA, Drosten C, Pöhlmann S. 2020. SARS-CoV-2 cell entry depends on ACE2 and TMPRSS2 and is blocked by a clinically proven protease inhibitor. Cell 181:271–280. doi:10.1016/j.cell.2020.02.052 32142651 PMC7102627

[2] Takeda M. 2022. Proteolytic activation of SARS-CoV-2 spike protein. Microbiol Immunol 66:15–23. doi:10.1111/1348-0421.12945 34561887 PMC8652499

[3] Plescia CB, David EA, Patra D, Sengupta R, Amiar S, Su Y, Stahelin RV. 2021. SARS-CoV-2 viral budding and entry can be modeled using BSL-2 level virus-like particles. J Biol Chem 296:100103. doi:10.1074/jbc.RA120.016148 33214224 PMC7832013

[4] Swann H, Sharma A, Preece B, Peterson A, Eldredge C, Belnap DM, Vershinin M, Saffarian S. 2020. Minimal system for assembly of SARS-CoV-2 virus like particles. Sci Rep 10:21877. doi:10.1038/s41598-020-78656-w 33318562 PMC7736577

[5] Xu R, Shi M, Li J, Song P, Li N. 2020. Construction of SARS-CoV-2 virus-like particles by mammalian expression system. Front Bioeng Biotechnol 8:862. doi:10.3389/fbioe.2020.00862 32850726 PMC7409377

[6] Hagemeijer MC, Monastyrska I, Griffith J, van der Sluijs P, Voortman J, van Bergen en Henegouwen PM, Vonk AM, Rottier PJM, Reggiori F, de Haan CAM. 2014. Membrane rearrangements mediated by coronavirus nonstructural proteins 3 and 4. Virology (Auckl) 458–459:125–135. doi:10.1016/j.virol.2014.04.027 PMC7111329 24928045

[7] Oudshoorn D, Rijs K, Limpens R, Groen K, Koster AJ, Snijder EJ, Kikkert M, Bárcena M. 2017. Expression and cleavage of middle east respiratory syndrome coronavirus nsp3-4 polyprotein induce the formation of double-membrane vesicles that mimic those associated with coronaviral RNA replication. MBio 8:e01658-17. doi:10.1128/mBio.01658-17 29162711 PMC5698553

[8] Angelini MM, Akhlaghpour M, Neuman BW, Buchmeier MJ. 2013. Severe acute respiratory syndrome coronavirus nonstructural proteins 3, 4, and 6 induce double-membrane vesicles. MBio 4:e00524-13. doi:10.1128/mBio.00524-13 PMC3747587 23943763

[9] Klein S, Cortese M, Winter SL, Wachsmuth-Melm M, Neufeldt CJ, Cerikan B, Stanifer ML, Boulant S, Bartenschlager R, Chlanda P. 2020. SARS-CoV-2 structure and replication characterized by in situ cryo-electron tomography. Nat Commun 11:5885. doi:10.1038/s41467-020-19619-7 33208793 PMC7676268

[10] Snijder EJ, Limpens R, de Wilde AH, de Jong AWM, Zevenhoven-Dobbe JC, Maier HJ, Faas F, Koster AJ, Bárcena M. 2020. A unifying structural and functional model of the coronavirus replication organelle: tracking down RNA synthesis. PLoS Biol 18:e3000715. doi:10.1371/journal.pbio.3000715 32511245 PMC7302735

[11] Wolff G, Limpens RWAL, Zevenhoven-Dobbe JC, Laugks U, Zheng S, de Jong AWM, Koning RI, Agard DA, Grünewald K, Koster AJ, Snijder EJ, Bárcena M. 2020. A molecular pore spans the double membrane of the coronavirus replication organelle. Science 369:1395–1398. doi:10.1126/science.abd3629 32763915 PMC7665310

[12] Cortese M, Lee J-Y, Cerikan B, Neufeldt CJ, Oorschot VMJ, Köhrer S, Hennies J, Schieber NL, Ronchi P, Mizzon G, et al.. 2020. Integrative imaging reveals SARS-CoV-2-induced reshaping of subcellular morphologies. Cell Host Microbe 28:853–866. doi:10.1016/j.chom.2020.11.003 33245857 PMC7670925

[13] Klumperman J, Locker JK, Meijer A, Horzinek MC, Geuze HJ, Rottier PJ. 1994. Coronavirus M proteins accumulate in the Golgi complex beyond the site of virion budding. J Virol 68:6523–6534. doi:10.1128/JVI.68.10.6523-6534.1994 8083990 PMC237073

[14] Scherer KM, Mascheroni L, Carnell GW, Wunderlich LCS, Makarchuk S, Brockhoff M, Mela I, Fernandez-Villegas A, Barysevich M, Stewart H, Suau Sans M, George CL, Lamb JR, Kaminski-Schierle GS, Heeney JL, Kaminski CF. 2022. SARS-CoV-2 nucleocapsid protein adheres to replication organelles before viral assembly at the Golgi/ERGIC and lysosome-mediated egress. Sci Adv 8:eabl4895. doi:10.1126/sciadv.abl4895 34995113 PMC10954198

[15] Tooze J, Tooze SA, Fuller SD. 1987. Sorting of progeny coronavirus from condensed secretory proteins at the exit from the trans-Golgi network of AtT20 cells. J Cell Biol 105:1215–1226. doi:10.1083/jcb.105.3.1215 2821011 PMC2114808

[16] Stertz S, Reichelt M, Spiegel M, Kuri T, Martínez-Sobrido L, García-Sastre A, Weber F, Kochs G. 2007. The intracellular sites of early replication and budding of SARS-coronavirus. Virology (Auckl) 361:304–315. doi:10.1016/j.virol.2006.11.027 PMC7103305 17210170

[17] Ulasli M, Verheije MH, de Haan CAM, Reggiori F. 2010. Qualitative and quantitative ultrastructural analysis of the membrane rearrangements induced by coronavirus. Cell Microbiol 12:844–861. doi:10.1111/j.1462-5822.2010.01437.x 20088951 PMC7159092

[18] Lavi E, Wang Q, Weiss SR, Gonatas NK. 1996. Syncytia formation induced by coronavirus infection is associated with fragmentation and rearrangement of the Golgi apparatus. Virol (Auckl) 221:325–334. doi:10.1006/viro.1996.0382 PMC7131612 8661443

[19] Mendonça L, Howe A, Gilchrist JB, Sheng Y, Sun D, Knight ML, Zanetti-Domingues LC, Bateman B, Krebs AS, Chen L, Radecke J, Li VD, Ni T, Kounatidis I, Koronfel MA, Szynkiewicz M, Harkiolaki M, Martin-Fernandez ML, James W, Zhang P. 2021. Correlative multi-scale cryo-imaging unveils SARS-CoV-2 assembly and egress. Nat Commun 12:4629. doi:10.1038/s41467-021-24887-y 34330917 PMC8324836

[20] Saraste J, Enyioko M, Dale H, Prydz K, Machamer C. 2022. Evidence for the role of Rab11-positive recycling endosomes as intermediates in coronavirus egress from epithelial cells. Histochem Cell Biol 158:241–251. doi:10.1007/s00418-022-02115-y 35604431 PMC9124743

[21] Ghosh S, Dellibovi-Ragheb TA, Kerviel A, Pak E, Qiu Q, Fisher M, Takvorian PM, Bleck C, Hsu VW, Fehr AR, Perlman S, Achar SR, Straus MR, Whittaker GR, de Haan CAM, Kehrl J, Altan-Bonnet G, Altan-Bonnet N. 2020. β-coronaviruses use lysosomes for egress instead of the biosynthetic secretory pathway. Cell 183:1520–1535. doi:10.1016/j.cell.2020.10.039 33157038 PMC7590812

[22] Eymieux S, Uzbekov R, Rouillé Y, Blanchard E, Hourioux C, Dubuisson J, Belouzard S, Roingeard P. 2021. Secretory vesicles are the principal means of SARS-CoV-2 egress. Cells 10:2047. doi:10.3390/cells10082047 34440816 PMC8393858

[23] Sergio MC, Ricciardi S, Guarino AM, Giaquinto L, De Matteis MA. 2024. Membrane remodeling and trafficking piloted by SARS-CoV-2. Trends Cell Biol 34:785–800. doi:10.1016/j.tcb.2023.12.006 38262893

[24] Waters MG, Serafini T, Rothman JE. 1991. “Coatomer”: a cytosolic protein complex containing subunits of non-clathrin-coated Golgi transport vesicles. Nature New Biol 349:248–251. doi:10.1038/349248a0 1898986

[25] Letourneur F, Gaynor EC, Hennecke S, Démollière C, Duden R, Emr SD, Riezman H, Cosson P. 1994. Coatomer is essential for retrieval of dilysine-tagged proteins to the endoplasmic reticulum. Cell 79:1199–1207. doi:10.1016/0092-8674(94)90011-6 8001155

[26] Béthune J, Wieland FT. 2018. Assembly of COPI and COPII vesicular coat proteins on membranes. Annu Rev Biophys 47:63–83. doi:10.1146/annurev-biophys-070317-033259 29345989

[27] Lontok E, Corse E, Machamer CE. 2004. Intracellular targeting signals contribute to localization of coronavirus spike proteins near the virus assembly site. J Virol 78:5913–5922. doi:10.1128/JVI.78.11.5913-5922.2004 15140989 PMC415842

[28] McBride CE, Li J, Machamer CE. 2007. The cytoplasmic tail of the severe acute respiratory syndrome coronavirus spike protein contains a novel endoplasmic reticulum retrieval signal that binds COPI and promotes interaction with membrane protein. J Virol 81:2418–2428. doi:10.1128/JVI.02146-06 17166901 PMC1865919

[29] Koch J, Uckeley ZM, Doldan P, Stanifer M, Boulant S, Lozach PY. 2021. TMPRSS2 expression dictates the entry route used by SARS-CoV-2 to infect host cells. EMBO J 40:e107821. doi:10.15252/embj.2021107821 34159616 PMC8365257

[30] Robinson MS. 2015. Forty Years of Clathrin-coated Vesicles. Traffic 16:1210–1238. doi:10.1111/tra.12335 26403691

[31] Mitrovic S, Ben-Tekaya H, Koegler E, Gruenberg J, Hauri HP. 2008. The cargo receptors Surf4, endoplasmic reticulum-Golgi intermediate compartment (ERGIC)-53, and p25 are required to maintain the architecture of ERGIC and Golgi. Mol Biol Cell 19:1976–1990. doi:10.1091/mbc.e07-10-0989 18287528 PMC2366877

[32] Prydz K, Saraste J. 2022. The life cycle and enigmatic egress of coronaviruses. Mol Microbiol 117:1308–1316. doi:10.1111/mmi.14907 35434857 PMC9321882

[33] Popoff V, Adolf F, Brügger B, Wieland F. 2011. COPI budding within the Golgi stack. Cold Spring Harb Perspect Biol 3:a005231. doi:10.1101/cshperspect.a005231 21844168 PMC3220356

[34] Gomez-Navarro N, Miller E. 2016. Protein sorting at the ER-Golgi interface. J Cell Biol 215:769–778. doi:10.1083/jcb.201610031 27903609 PMC5166505

[35] de Wilde AH, Wannee KF, Scholte FEM, Goeman JJ, Ten Dijke P, Snijder EJ, Kikkert M, van Hemert MJ. 2015. A kinome-wide small interfering RNA screen identifies proviral and antiviral host factors in severe acute respiratory syndrome coronavirus replication, including double-stranded RNA-activated protein kinase and early secretory pathway proteins. J Virol 89:8318–8333. doi:10.1128/JVI.01029-15 26041291 PMC4524262

[36] Dey D, Singh S, Khan S, Martin M, Schnicker NJ, Gakhar L, Pierce BG, Hasan SS. 2022. An extended motif in the SARS-CoV-2 spike modulates binding and release of host coatomer in retrograde trafficking. Commun Biol 5:115. doi:10.1038/s42003-022-03063-y 35136165 PMC8825798

[37] Li Q, Liu Y, Zhang L. 2022. Cytoplasmic tail determines the membrane trafficking and localization of SARS-CoV-2 spike protein. Front Mol Biosci 9:1004036. doi:10.3389/fmolb.2022.1004036 36225258 PMC9548995

[38] Bergner T, Zech F, Hirschenberger M, Stenger S, Sparrer KMJ, Kirchhoff F, Read C. 2022. Near-native visualization of SARS-CoV-2 induced membrane remodeling and virion morphogenesis. Viruses 14:2786. doi:10.3390/v14122786 36560790 PMC9784144

[39] Mironov AA, Savin MA, Beznoussenko GV. 2023. COVID-19 biogenesis and intracellular transport. IJMS 24:4523. doi:10.3390/ijms24054523 36901955 PMC10002980

[40] Matsuyama S, Nao N, Shirato K, Kawase M, Saito S, Takayama I, Nagata N, Sekizuka T, Katoh H, Kato F, Sakata M, Tahara M, Kutsuna S, Ohmagari N, Kuroda M, Suzuki T, Kageyama T, Takeda M. 2020. Enhanced isolation of SARS-CoV-2 by TMPRSS2-expressing cells. Proc Natl Acad Sci U S A 117:7001–7003. doi:10.1073/pnas.2002589117 32165541 PMC7132130

[41] Muramoto Y, Takahashi S, Halfmann PJ, Gotoh S, Noda T, Kawaoka Y. 2023. Replicative capacity of SARS-CoV-2 omicron variants BA.5 and BQ.1.1 at elevated temperatures. Lancet Microbe 4:e486. doi:10.1016/S2666-5247(23)00100-3 37105204 PMC10124997

[42] Reed LJ, Muench H. 1938. A simple method of estimating fifty per cent endpoints. Am J Epidemiol 27:493–497. doi:10.1093/oxfordjournals.aje.a118408

[43] Kremer JR, Mastronarde DN, McIntosh JR. 1996. Computer visualization of three-dimensional image data using IMOD. J Struct Biol 116:71–76. doi:10.1006/jsbi.1996.0013 8742726

[44] Noda T, Sagara H, Yen A, Takada A, Kida H, Cheng RH, Kawaoka Y. 2006. Influenza A virus. Nat New Biol 439:490–492. doi:10.1038/nature04378 16437116

