# Supplementary material for: Coatomer complex I is required for the transport of SARS-CoV-2 progeny virions from the endoplasmic reticulum-Golgi intermediate compartment
Source: mBio. 2024 Nov 29;16(1):e03331-24. doi: 10.1128/mbio.03331-24 (PMC11708035; doi:10.1128/mbio.03331-24)
Supplement: Supplemental Legends — Legends for Movies S1 to S8. [file mbio.03331-24-s0001.docx]

**Supplementary Figure legends**

**Movie S1.** 3D image of a SARS-CoV-2-infected VeroE6/TMPRSS2 cell reconstructed using SEM array tomography.

**Movie S2.** 3D image of ERGICs containing progeny virions reconstructed using SEM array tomography. 3D rendering was performed for ERGIC vesicles/tubules (light blue, blue, and purple), budding virions (orange), and budded virions (yellow).

**Movie S3.** 3D image of a multiple virion-containing large vacuole reconstructed using SEM array tomography. 3D rendering was performed for virion-containing large vacuole (light green), budding virions (orange), and budded virions (yellow).

**Movie S4.** 3D image of single virion-containing small vesicles reconstructed using SEM array tomography. 3D rendering was performed for single virion-containing small vesicles (light blue), and budded virions (yellow).

**Movie S5.** Electron tomographic image of small vesicles/pits coated with proteins that are located beneath the plasma membrane of virus-infected cell.

**Movie S6.** 3D image of a multiple virions-containing large vacuole that is located beneath the plasma membrane of the virus-infected cell, reconstructed using SEM array tomography. 3D rendering was performed for virion-containing large vacuole (light blue), budding virions (orange), and budded virions (yellow).

**Movie S7.** Electron tomographic image of multiple virion-containing vesicles located beneath the plasma membrane of virus-infected cell. Some virion-containing vesicles have protrusion coated with proteins.

**Movie S8.** 3D image of a multiple virion-containing lysosome that is fused with the plasma membrane of virus-infected cell, reconstructed using SEM array tomography. 3D rendering was performed for virion-containing lysosome (light blue), degraded cellular components (gray), and budded virions (yellow).
